# Wild Western Lowland Gorillas Signal Selectively Using Odor

**DOI:** 10.1371/journal.pone.0099554

**Published:** 2014-07-09

**Authors:** Michelle Klailova, Phyllis C. Lee

**Affiliations:** 1 Division of Psychology, Behaviour, Evolution and Research Group, School of Natural Sciences, University of Stirling, Stirling, United Kingdom; 2 Departamento de Antropologia, Centro de Administração e Politicas Públicas, Instituto Superior de Ciências Sociais e Políticas, Universidade de Lisboa, Lisboa, Portugal; 3 Wildlife Conservation Research Unit, Department of Zoology, University of Oxford, The Recanati-Kaplan Centre, Tubney, United Kingdom; University of Florence, Italy

## Abstract

Mammals communicate socially through visual, auditory and chemical signals. The chemical sense is the oldest sense and is shared by all organisms including bacteria. Despite mounting evidence for social chemo-signaling in humans, the extent to which it modulates behavior is debated and can benefit from comparative models of closely related hominoids. The use of odor cues in wild ape social communication has been only rarely explored. Apart from one study on wild chimpanzee sniffing, our understanding is limited to anecdotes. We present the first study of wild gorilla chemo-communication and the first analysis of olfactory signaling in relation to arousal levels and odor strength in wild apes. If gorilla scent is used as a signaling mechanism instead of only a sign of arousal or stress, odor emission should be context specific and capable of variation as a function of the relationships between the emitter and perceiver(s). Measured through a human pungency scale, we determined the factors that predicted extreme levels of silverback odor for one wild western lowland gorilla (*Gorilla gorilla gorilla*) group silverback. Extreme silverback odor was predicted by the presence and intensity of inter-unit interactions, silverback anger, distress and long-calling auditory rates, and the absence of close proximity between the silverback and mother of the youngest infant. Odor strength also varied according to the focal silverback's strategic responses during high intensity inter-unit interactions. Silverbacks appear to use odor as a modifiable form of communication; where odor acts as a highly flexible, context dependent signaling mechanism to group members and extra-group units. The importance of olfaction to ape social communication may be especially pertinent in Central African forests where limited visibility may necessitate increased reliance on other senses.

## Introduction

A growing body of evidence indicates that mammals, like social insects, use their sense of smell in subtle and intricate ways [1]. Olfactory communication is said to occur when a sender transmits a chemical signal whose properties affect the behavior or physiology of a receiver [1], [2].

Although chemical cues are emerging as key mechanisms used in mammalian recognition, mate choice, resource defence, and competition [1], their influence on great ape sociality remains controversial and vastly unexplored [3]. Two main factors have contributed to this controversy and dearth of information. Firstly, great apes are considered to be highly microsomatic since (a) relative olfactory brain size has decreased and olfactory receptor pseudogene numbers have increased over primate evolutionary history, and; (b) great apes appear to lack a functional vomeronasal organ (VNO) implicated in the pheromone processing of other mammals [4], [5], [6]. Secondly, logistical difficulties involved in studying ape chemo-communication - particularly in wild environments involving unhabituated apes - have often prevented or dissuaded researchers from collecting appropriate data.

Nevertheless, research on human chemo-communication is growing despite their microsomatic label. Evidence suggests that human odor can signal identity, quality, mood, and status [7], [8], [9], [10]. Humans have even been shown to discriminate odors between congenic mice at the major histocompatibility complex (MHC) level [11]; a chromosomal region containing genes that play a role in immunological recognition [12]. Therefore, chemical processing can occur without a functional VNO [3] and may be more dependent on the biological relevance of the odor rather than olfactory brain size and receptor numbers [13].

As interest in and evidence for human chemo-communication mounts, investigative aims should broaden to include comparative models among other great apes. Wild data collection on great ape olfactory communication – while still very challenging – is now becoming feasible due to advances in technology and the increased availability of habituated wild great ape groups. Here, we present the first study of wild gorilla chemo-communication and we explore the factors that predict odor production and intensity in western lowland gorilla adult males (also known as silverbacks).

Western lowland gorilla females and offspring live in groups with one protector adult male [14]. Adult females emigrate directly to other groups by choice during inter-unit interactions (hereafter also ‘interactions’) [15]. Upon emigration, males commonly range solitarily until females are acquired [14]. As predicted for polygnous species and for one of the most sexually dimorphic primates, male-male competition for, or in defense of, female gorillas is fierce [16] and one gorilla group's range may contain several solitary males in search of females [14], [15], [17]. Though often limited to threat displays, inter-unit interactions can lead to silverback death and infanticide [18].

Marked within-sex differences of secondary sexual characters in male gorillas advertize strength, quality and fighting ability [16], [19]. The development of secondary sexual characters is androgen - primarily testosterone - dependent [20]. Androgen dependent traits are thought to honestly advertize male quality, since high androgen levels may pose an immunocompetence handicap [21], [22]. In western lowland gorillas, large gluteal muscles, saggital crests and body length are androgen dependent traits reflective of male reproductive success [16], [19]. Moreover, the tendency to dominate (using both physicality and social intelligence) through achieving and maintaining high status is also thought to be testosterone dependent and thus an honest indicator of male quality [23]. Indeed, dominant adult male mountain gorillas (*Gorilla beringei beringei*) which live in heterosexual multi-male groups have higher testosterone levels than their subordinates [24].

Androgen-derived steroids are also involved in chemo-signaling [1], [9], [25]. In humans, the main region implicated in chemo-communication is the axillary region [9]. The axillary region contains a high density of secretory glands which exude androstenes and other androgen-derived compounds. Once acted upon by bacteria, these secretions are responsible for the characteristic odor of adult males [9]. Of all non-human primates, only chimpanzees and gorillas have a comparable density of secretory glands in the axillary region to humans [26], [27], [28]. As androstene production is subject to sexual selection and likely metabolically linked to testosterone [9], body odor in apes may communicate genomic and metabolic information that act as honest signals of quality, status, and identity [1], [21], [25]. This may be especially relevant for the highly odorous adult male gorilla.

Ape studies in captivity [29], [30], [31], [32], [33], chimpanzee and orangutan anecdotes in the wild [34], [35], [36], and one study on wild chimpanzee sniffing [35] show that non-human apes (like humans) can discriminate between different odors. In wild gorillas, anecdotal reports on the presence and potential importance of gorilla adult male scent date back to 1958, detailing an unmistakably pungent odor [27], [37], [38], [39], [40], [41], [42], which some [e.g. Fossey] feel is supposedly emitted in “stress” or “fear” [27]. Humans can reliably identify individual western lowland gorillas - particularly the “intense” smelling silverback - by their odor [43]. Thus (a) gorillas produce individually identifiable odors, which are a precursor for olfactory communication, and; (b) gorillas should be able to distinguish individuals by odor since the more microsomatic human can do so with high certainty.

Here, we examine the relationship between silverback arousal levels and silverback odor production in high risk contexts for one habituated western lowland group led by their only silverback, Makumba. Although the analyses are mainly exploratory, we make the following broad predictions: (1) while fear odor can communicate emotion in mammals, alerting kin to danger, priming flight and vigilance responses, and enhancing cognitive performance (e.g. deer [44], humans [45]), gorilla odor has broader functions and represents more than a sign of arousal, stress, or fear, and; (2) silverbacks use odor and modifiable odor pungency/intensity as a signaling mechanism to communicate to group members and extra-group units.

Results suggest that silverbacks may use context specific chemo-signals to moderate the social behaviors of other gorillas, which corroborates our broad predictions, provides evidence towards a comparative model of olfactory communicative effects in hominoids, and illustrates the necessity for further research on chemo-communication in apes.

## Methods

### Ethics statement

Ethical clearance for this study was granted by the University of Stirling, Division of Psychology, Ethics Committee. Permits and approvals for fieldwork were obtained from the Government of the Central African Republic (CAR) and the World Wildlife Fund - Dzanga Sangha Protected Areas Complex (DSPA). The DSPA covers over 4, 500 km^2^ and consists of the Dzanga and Ndoki Sector of the Dzanga-Ndoki National Park, as well as the Dzanga-Sangha Forest Reserve. The DSPA forms part of the Sangha Tri National Complex of protected areas. Data was collected in the Dzanga sector of the DSPA. All data collected were purely observational. Observer teams took every precaution to follow Best Practice guidelines for minimizing stress to the focal group and for minimizing the risk of human-gorilla disease transmission [46].

### Study site and focal gorilla group

Research was conducted for 12 months from January 2007 at the Bai Hokou Primate Habituation Camp (2°50′N, 16°28′E). The Makumba gorilla group consisted of 13 individuals: one silverback, three adult females, two subadults, one blackback, four juveniles, and two infants (plus one birth in December 2007 bringing the group size to 14 individuals; Table S1 in File S1). The Makumba group was followed from nest-to-nest by a team of trackers (range 2–4) and researchers (range 1–3).

### Odor intensity ratings

A subjective silverback odor intensity rating was defined as (0) none, where no odor was detected; (1) low, where slight odor was detected not stronger than the smell of surrounding vegetation; (2) high, where odor detected was stronger than the smell of surrounding vegetation, and; (3) extreme, where odor detected was extremely conspicuous and the only element that could be smelled in the surrounding air. Data were recorded by two raters. Raters did not sensitize to odors over time (Table S2 in File S1). Due to the novel and exploratory nature of this research and in order to provide the necessary baseline for gorilla chemo-communication, this article focuses mostly on Makumba's extreme odor emission. In future analyses we aim to incorporate the effects of all other odor intensities on social responses.

### Data scans

Instantaneous scans [47] of the activity and proximity of the silverback and his nearest neigbors (within five meters) were taken every 10 minutes unless visibility was briefly lost, at which point scanning resumed at the point of re-contact. Odor intensity ratings along with detailed behavioral and ecological information were recorded during each scan (Table S3 in File S1). If an odor was detected outside scan times, its intensity rating along with additional environmental and behavioral information were recorded as *ad libitum* data (Table S3 in File S1). In order to minimize any effects of a lingering smell, five minutes must have elapsed between any odor recording.

Data on the identity of all individuals within visual contact of the silverback (hereafter ‘roll call’) were collected at hourly intervals throughout observation periods. Each roll call allowed for an assessment of individuals who were both in close proximity and further away - though likely still within auditory, olfactory and visual range - of the silverback. Analyses of individuals in proximity to the silverback are thus based on both specific nearest-neighbor data within five meters and general hourly roll call data. When proximity data collected solely from roll calls are reported, we refer to the individual(s) as being ‘in the vicinity of’ or ‘close to’ the silverback.

### Auditory signals

In addition to instantaneous scans, we took continuous written records of all auditory signals made by the focal silverback (followed 100% of the time) and of any gorillas in his presence (within human earshot). Auditory signaling rates of individuals far from Makumba may thus be under-represented. However, major shifts in group dynamics were almost always centered upon the focal silverback. Therefore, general auditory signaling patterns in relation to silverback-group communication should still be accurately reflected in the data set.

Auditory signals were defined as any sound made by a gorilla, either orally or via non-oral auditory displays (i.e. ground-slapping, tree-breaking, chest-beating) and were categorized by function, age-sex class, and presumed arousal levels. Anger or distress signals represented high arousal levels and involved barking, charging, screaming, and non-oral auditory displays. Long-calling signals - often used in long-distance inter or extra-unit communication - also represented high arousal levels and consisted of chest-beating and/or hooting. Soft signals - used in close contact intra-unit communication - represented low arousal levels and involved belching and soft whinnys/neighs. Medium arousal was exhibited through a variety of auditory signals such as play grunting, juvenile hand clapping, humming, and other types of whinnys/neighs. For detailed definitions of auditory signals in gorillas see [48], [49], [50], [51].

### Inter-unit interactions

Intensity of an inter-unit interaction between the focal group silverback and another unit (either another group silverback or a solitary silverback) was rated as (1) low, where (a) dung and vegetation traces, ranging behaviour, and nests indicated a potential interaction but the focal group behaved normally when observer contact was made, or, (b) the focal silverback behaved in a manner which suggested the presence of another unit, although the other unit was neither heard nor seen by the observer; (2) medium, where an auditory exchange occurred between the focal group and the extra-group unit, and; (3) high, where auditory and visual exchange between the focal group and extra-group unit occurred, with or without physical contact.

### Analysis

Final analyses were conducted on (a) 3, 252 instantaneous scans, where each scan was associated with one odor rating; (b) 1, 053 additional *ad libitum* odor rating data points with associated *ad libitum* behavioral and environmental information, and; (c) 22, 343 written auditory signal recordings of the silverback and of all other individuals in his presence.

Analysis of silverback activities ensured that there were no autocorrelations between successive scans (Figure S1 in File S1). Successive smell ratings were also statistically independent from one another (Figure S2 in File S1). Nevertheless, data were grouped for analysis into 258 morning or afternoon sessions to limit any effects of dependence between successive auditory records. Hourly rates, relative to the number of observation minutes in each session, were calculated for all auditory signals. Odor intensity proportions were calculated out of total smell recordings for each session. The proportion of hourly presence (using roll call) of each individual within the vicinity of the silverback was calculated for each session. For additional information on data grouping see Table S4 in File S1.

To measure auditory signaling changes during inter-unit interactions, auditory data were compared between periods of one hour before to one hour after an interaction. Inter-unit interactions were further grouped into those with quiet and loud silverback responses. Quiet and loud responses were determined from the frequency distribution of Makumba's hourly rates of loud auditory signals (anger, distress and long-call signals). An hourly rate in the first quartile was categorized as a ‘quiet’ response (<2.99, range 0–2.67). Hourly rates in the remaining quartiles were categorized as ‘loud’ responses (>3.00, range 3.69–11.51). Quiet and loud response categorization corroborated with *ad libitum* notes written during each high intensity interaction (Table S5 in File S1).

Data were analyzed using SPSS Statistics Version 18 (IBM Corp). Probabilities are two tailed, and considered significant when *p*<0.05. The Forward Stepwise Logistic Regression was the main analytical tool, as there were no *a priori* predictions for predictor variable input order. All logistic regression analyses were conducted on non-transformed data, but data were square-root transformed for other tests where normalization was necessary. Highly correlated predictors (*r*>0.800) were excluded from analyses or analyzed separately. To assess the robustness of the observed effects, we present significance values as well as the proportion of variance explained by the model fit.

Similar to Schaller's [40] descriptions, extreme smells could be recognized further than 100 meters from the emitter. As there was no *a priori* knowledge for which factors may determine and effect the detection of extreme silverback odor, the initial logistic regression models contained up to 45 exploratory predictors, all of which could potentially influence silverback scent production or bias scent detection. These predictor variables were environmental (e.g. rainfall, wind), bio-geographical (e.g. forest density), human-influenced (e.g. human-directed aggression, observer-silverback distance), auditory (e.g. anger and distress), and behavioral (e.g. nearest-neighbor numbers, roll call, group spread, inter-unit interaction presence, silverback activity, silverback body position) (Table S3 in File S1 and Table S6 in File S1).

Once exploratory analyses were conducted, logistic models were re-run using only the final variables predicted from each initial model. Results of the final (i.e. re-run) models are reported here. Nagelkerke *R^2^* is reported and a Bonferroni correction (N-1 design for repeat tests of different queries on the same data set) has been applied to the overall logistic models relating to odor intensity (i.e. low, high, extreme) which were considered significant when *p*<0.025.

Since hourly roll call collection only began in March, the first two months of scan data (*n* = 66 sessions) were dropped from logistic models that included an individual's hourly roll call as one of the final predictors. Nonetheless, the addition of roll call into logistic analyses resulted in stronger models predicting more of the variance in the outcome. Hourly roll call potentially represents a more accurate way to (a) capture the movements of each gorilla, and; (b) assess overall proximity relationships with the silverback.

## Results

**Figure 1:**
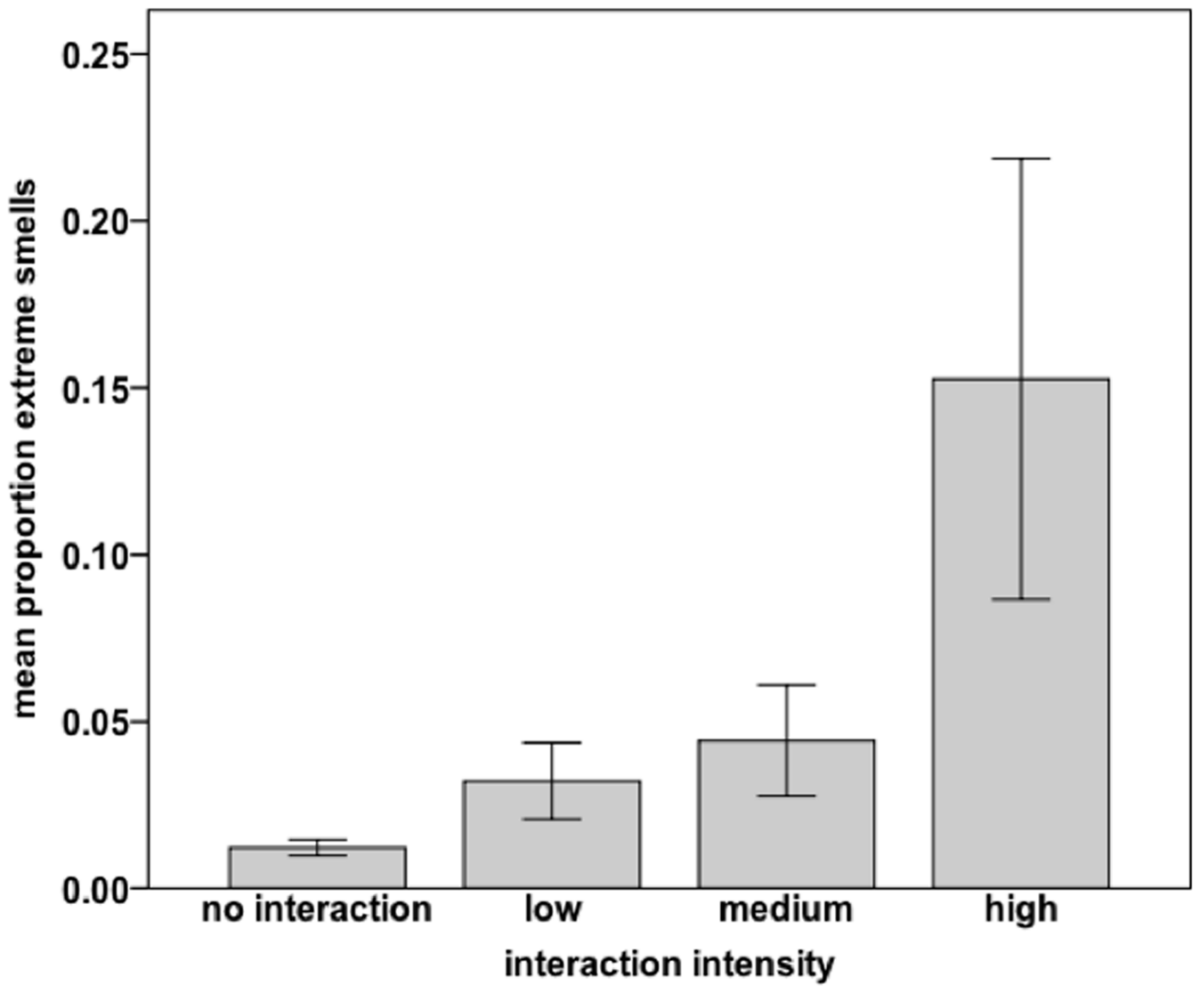
The predictive effect of inter-unit interaction intensity on silverback extreme smell. For no interactions *n* = 212. For low intensity interactions *n* = 21 (one interaction omitted due to missing data). For medium intensity interactions *n* = 11. For high intensity interactions *n* = 10 (one interaction omitted due to missing data). Error bars: ± 1s.e.

**Figure 2:**
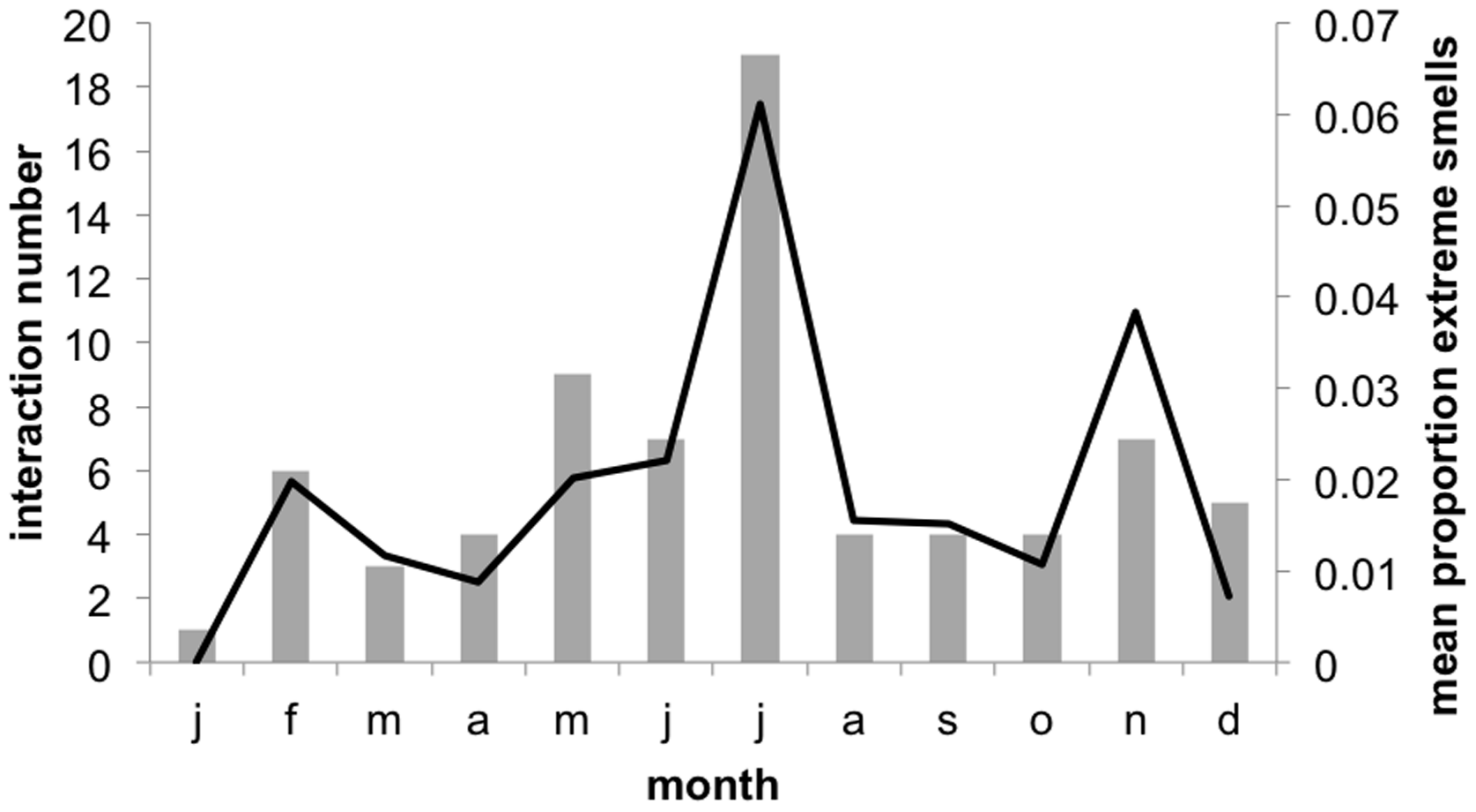
Total monthly interaction rates in relation to silverback extreme smell. A total of *n* = 79 interactions occurred in 2007.

Behavior and odor data were available for 44 of the 79 total inter-unit interactions recorded from 365 nest-to-nest follows and night traces. Extreme smells were predicted by (1) the silverback's auditory anger and distress signaling rates (R^2^ = 0.110); (2) inter-unit interaction occurrence (R^2^ = 0.060); (3) absence of close proximity between the mother of the youngest infant (Bombe) and the silverback (R^2^ = 0.054), and; (4) the silverback's auditory long-calling rates (R^2^ = 0.034); Table 1, Figure S3 in File S1, SPSS Data Set S1 http://dx.doi.org/10.6084/m9.figshare.1051748. Additionally, as interaction intensity increased, the emission of extreme odors significantly increased (logistic regression where *β* = 0.778, s.e. = 0.248, Wald = 9.811, df = 1, 4, R*^2^* = 0.061, Exp(*β*) = 2.177, *p* = 0.002, *n* = 184; Figure 1). Moreover, the total monthly number of interactions (*n* = 79) explained 88% of the monthly variance in the emission of extreme smell (*r* = 0.937, *p*<0.001, *n* = 12; partial correlation controlled for mean monthly rainfall which can attenuate odors [52], and silverback total auditory signaling rates; Figure 2, Figure S4 in File S1, SPSS Data Set S2 http://dx.doi.org/10.6084/m9.figshare.1051746).

**Table 1:** Predictors of silverback (sb) extreme smell.

predictors	*β*	s.e.	wald	df	model R^2^ at each step	exp(*β*)	sig
constant	−2.023	0.500	16.372	1	-	0.132	<0.001
sb anger and distress rate	0.788	0.294	7.008	1	.11	2.177	0.008
interaction presence	1.327	0.457	8.434	1	.17	3.770	0.004
bombe hourly presence by proportion	−1.489	0.607	6.014	1	.224	0.226	0.014
sb chest-beat and/or long-call rate	0.231	0.108	4.606	1	0.258	1.260	0.032

Logistic regression where overall model *X* ^2^ = 33.83, df = 4, R^2^ = 0.258, *p*<0.001, *n* = 185).

**Figure 3:**
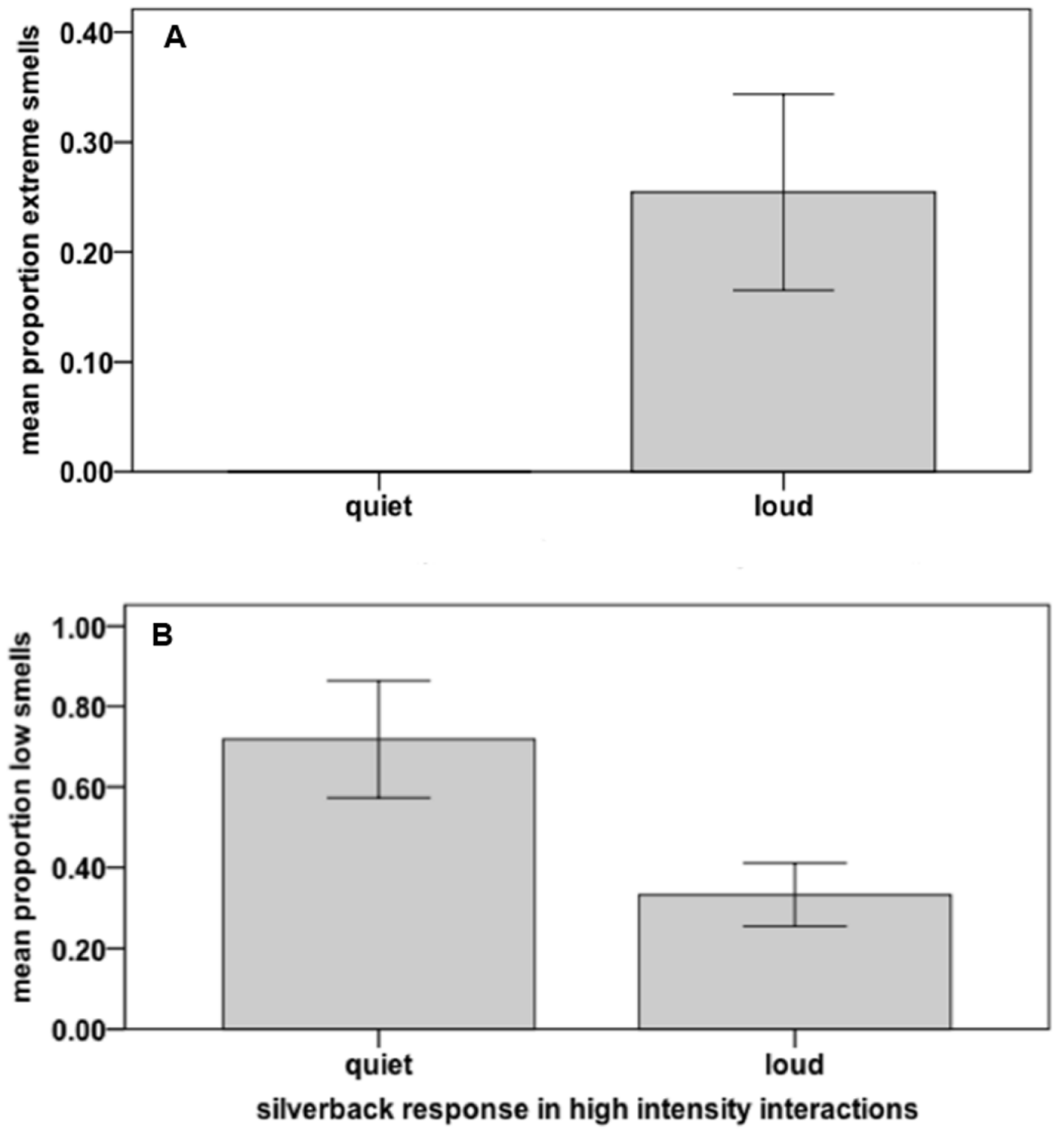
Silverback auditory responses during high intensity inter-unit interactions in relation to his extreme and low smells. For quiet response *n* = 4. For loud response *n* = 6; one loud interaction was omitted due to missing data. Error bars: ± 1s.e. Figure 3A relates to silverback extreme smell. Figure 3B relates to silverback low smell.

During the 11 high intensity inter-unit interactions for which auditory reactions were available, Makumba responded by (a) staying quiet, and fleeing or hiding with his group (*n* = 4), or; (b) making his position known through loud auditory signals with visual threat displays that sometimes escalated into physical contact (*n* = 7). Extreme silverback smells during high intensity interactions were not detected in any of the quiet responses (F = 27.35, df = 1, 7, *p* = 0.001, R^2^ = 0.739, *n* = 10; hierarchical ANOVA controlling for silverback total auditory rates; Figure 3A). However, significantly more low silverback smells were recorded during the quiet responses than during the loud responses (F = 6.91, df = 1, 7, *p* = 0.034, R^2^ = 0.353, and *n* = 10; hierarchical ANOVA controlling for silverback total auditory rates; Figure 3B, SPSS Data Set S3 http://dx.doi.org/10.6084/m9.figshare.1051747).

Low intensity odors were also more frequent as the number of neighbors within five meters of the silverback increased (logistic regression where *β* = 1.939, s.e. = 0.857, Wald = 5.116, df = 1, 4, R*^2^* = 0.039, Exp(*β*) = 6.951, *p* = 0.024, *n* = 186; Table S7 in File S1). This relationship was predicted irrespective of interaction presence or intensity.

## Discussion

At first glance extreme scent appears to act solely as an acute indicator of arousal increasing and decreasing rapidly in response to risky contexts, and thus may be viewed only as a by-product of fear or stress as suggested by Fossey [27]. If this were the case, then extreme odors should be present during all high intensity, high arousal interactions, regardless of the silverback's loud or quiet response. However, the ‘silence’ associated with a quiet response also extended to smell production. This effect presents compelling evidence that alarm signaling [1], [27], [37], [40], [42] is not the only function of extreme odor production in silverbacks, although the processing of different olfactory signals could indeed pass through similar neurological and biochemical networks as suggested for the human link in fear and rage-circuits [7]. Indeed, over 50 years ago Schaller [40] noted – though he was unable to determine the purpose or source of the odor - that on certain days he would smell silverback scent easily, while on others he would not smell any odor at all despite a breeze blowing in his direction.

During male-male competition, chemo-signals maintain territory, suppress mood and performance, intimidate rivals, and advertize status (e.g. humans [8], [9], [25]; non-human primates and other mammals [1], [2], [21], [53], [54], [55]). Like testosterone levels which respond to competition during periods of social instability (e.g. baboons [56], humans [57]), androgen-derived steroids involved in chemo-signaling are also responsive to changes in social context. For example: some insects and mammals completely suppress their chemo-signals due to loss or gain in status or when sneakily eavesdropping (e.g. cockroaches [58]; elephants [59]). However as of yet, almost nothing is known of the contribution of varying scent strengths (less intense to very intense) to chemo-communication.

Extreme chemo-signals in silverbacks appear to ‘yell’ at rival males or group members ranging far from the emitting silverback and thus should be clearly recognizable and minimally influenced by atmospheric surroundings. Since female gorillas transfer to other males through choice, solitary males may attempt to build trust by approaching females when the silverback is not near-by, or they may coerce females into transferring by committing infanticide [15]. Therefore, lack of knowledge of group member whereabouts can lead to dangerous consequences for western lowland group silverbacks, whose females often forage more than 500 meters away from their protector male [60]. This may explain Makumba's increase in extreme smell when his most vulnerable group member, the mother of the youngest infant, was not nearby.

Low intensity odors may instead be associated to close-contact intra-unit communication [2]. This may explain why low odors increased (a) as nearest-neighbor numbers to the silverback increased, and; (b) when the silverback remained quiet during high intensity interactions while hiding or fleeing with his group in close proximity. Low level odor emissions may be less costly (energetically), may enable the protector silverback to communicate with and re-assure group members, and may make it difficult for extra-unit silverback to eavesdrop [2], [54].

Our results suggest (a) that silverback odor strength can be ‘turned up’ or ‘turned down’ as well as ‘turned on’ or ‘turned off’ as a function of the context and relationship between the emitter and perceiver(s), and; (b) that varying odor intensities may communicate different context specific types of information.

Gorilla contests involve a combination of intense postural, facial and auditory threat displays that communicate status and strength to competing rivals and potential immigrant females [61]. Similar to other primates (e.g. lemurs [53], mandrills [21], humans [10], [62], [63]), gorilla chemo-signaling may be used in combination with other signals to reduce error when assessing mate quality and rival resource holding potential. Combining signal types is especially effective since unlike auditory and visual signals, olfactory signals can persist in the environment for long periods of time in absence of the sender, diffuse across large areas, and communicate in dark, dense and loud environments [2], [21], [64].

Olfactory signaling and the ability to fine tune odor strength may thus provide gorillas with an additional means of advertizing both fixed (identity, quality) and variable (status, mood, location) information [1]. Furthermore, gorilla chemo-communication - like human male competitive chemo-communication - may also play a role in suppressing competitor performance or in arousing mates [8], [25], [37].

Humans have retained many biochemical traits from ancestral taxa [65]. Understanding how these traits have evolved necessitates tracing its spread and understanding their function in other species [66]. We show for the first time in any non-human ape, that like humans, gorilla adult males appear to use highly context specific chemo-signals to moderate social behaviors.

## Supporting Information

Data Set S1 (SAV)

Data Set S2 (SAV)

Data Set S3 (SAV)

File S1 Contains supporting figures and tables. **Figure S1.** Cumulative rate of change plot in silverback activity budget. **Figure S2.** Cumulative rate of change plot in silverback smell. **Figure S3.** Bar graph of factors predicting silverback (sb) extreme smell. **Figure S4.** Additional information for total monthly interactions in relation to silverback extreme smell. **Table S1.** The Makumba group birthdates, ages and family trees. **Table S2.** Recorder non-sensitization to silverback smell. **Table S3.** Data recorded during instantaneous scans and *ad libitum* smell ratings. **Table S4.** Additional information on data groupings used in analysis. **Table S5.** Field note descriptions of quiet and loud silverback responses during high intensity interactions. **Table S6.** Predictors included in initial forward stepwise regressions. **Table S7.** Predictors of low silverback smell.
(PDF)
