# Supplementary material for: Wild Western Lowland Gorillas Signal Selectively Using Odor
Source: PLoS One. 2014 Jul 9;9(7):e99554. doi: 10.1371/journal.pone.0099554 (PMC4090154; doi:10.1371/journal.pone.0099554)
Supplement: File S1 — Contains supporting figures and tables. Figure S1. Cumulative rate of change plot in silverback activity budget. Figure S2. Cumulative rate of change plot in silverback smell. Figure S3. Bar graph of factors predicting silverback (sb) extreme smell. Figure S4. Additional information for total monthly interactions in relation to silverback extreme smell. Table S1. The Makumba group birthdates, ages and family trees. Table S2. Recorder non-sensitization to silverback smell. Table S3. Data recorded during instantaneous scans and ad libitum smell ratings. Table S4. Additional information on data groupings used in analysis. Table S5. Field note descriptions of quiet and loud silverback responses during high intensity interactions. Table S6. Predictors included in initial forward stepwise regressions. Table S7. Predictors of low silverback smell. (PDF) [file pone.0099554.s004.pdf]

Supporting Information File S1

Supporting Figures

Figure S1 Cumulative rate of change plot in silverback activity budget.

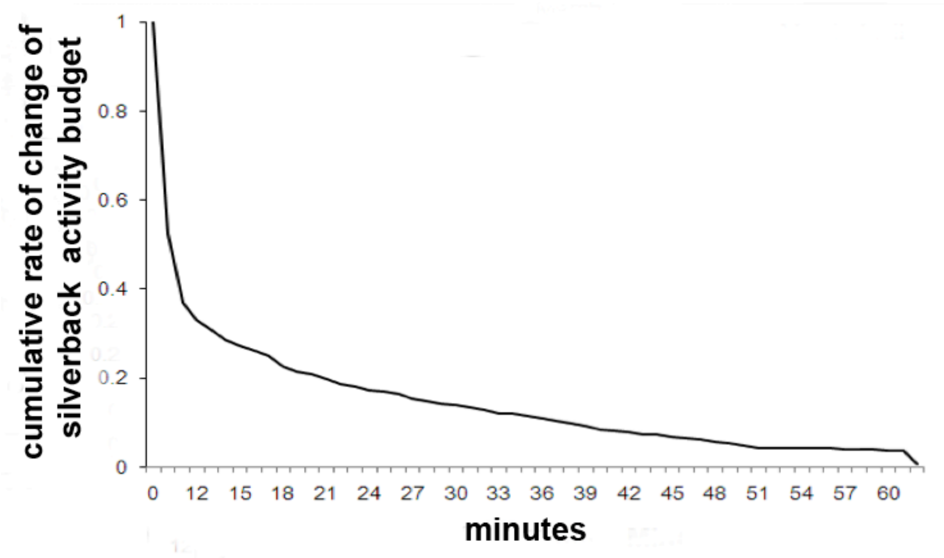

Since the silverback was the focus of the study and his behavior was likely to affect group behavior (or vice versa), Makumba's activity budget was analyzed to ensure there were no 'overall' autocorrelation issues. Silverback activities recorded a minimum of 10 minutes apart from each other can be considered statistically independent, as illustrated by the first break in slope of the cumulative plot analyzed from a baseline subset (March – April) of 2007 observation data. Other data often used in analyses (e.g. silverback-neighbor numbers) were also proven statistically independent [1].

Figure S2. Cumulative rate of change plot in silverback smell.

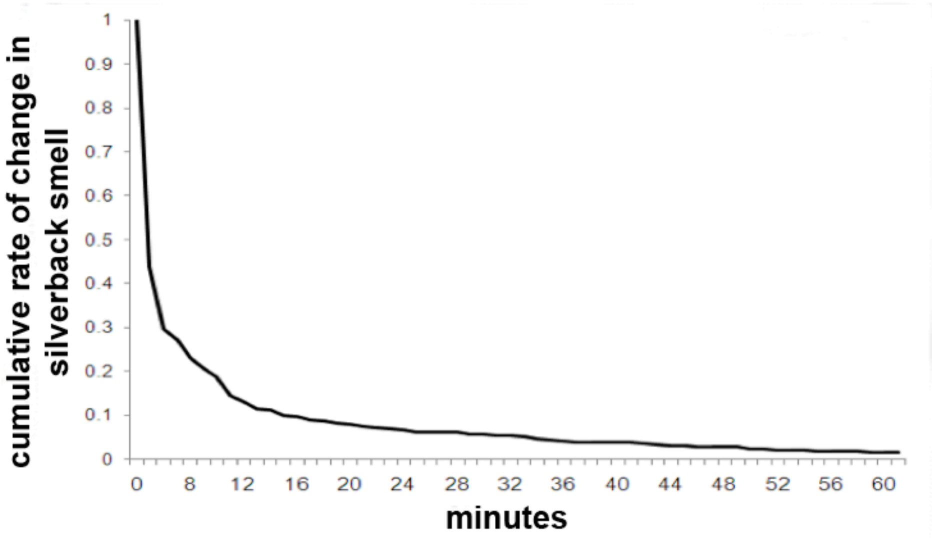

Silverback smell data points recorded five minutes apart are considered statistically independent, as illustrated by the first break in slope of the cumulative plot, analyzed from a baseline subset (March - April) of 2007 observation data.

Figure S3. Bar graph of factors predicting silverback (sb) extreme smell.

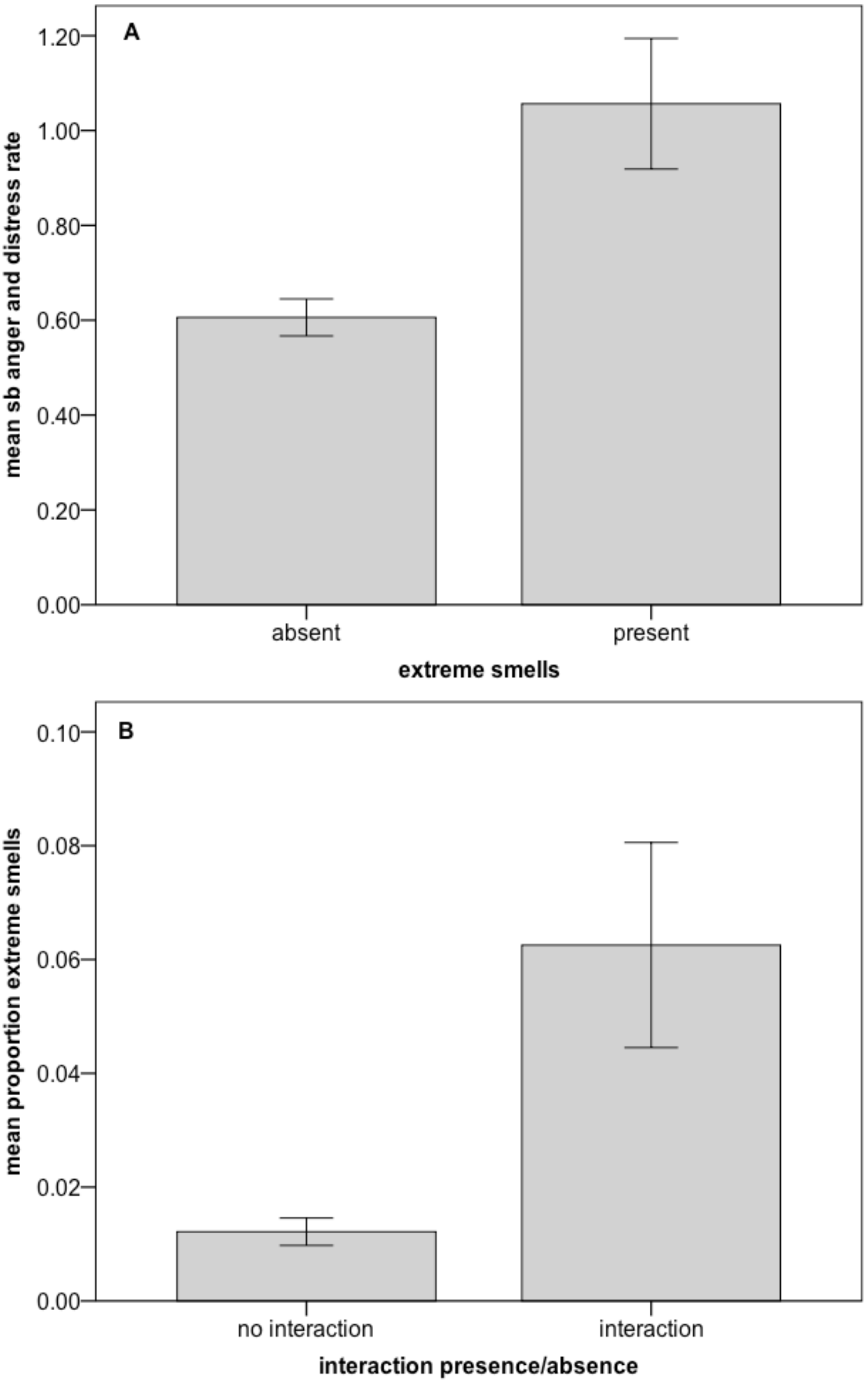

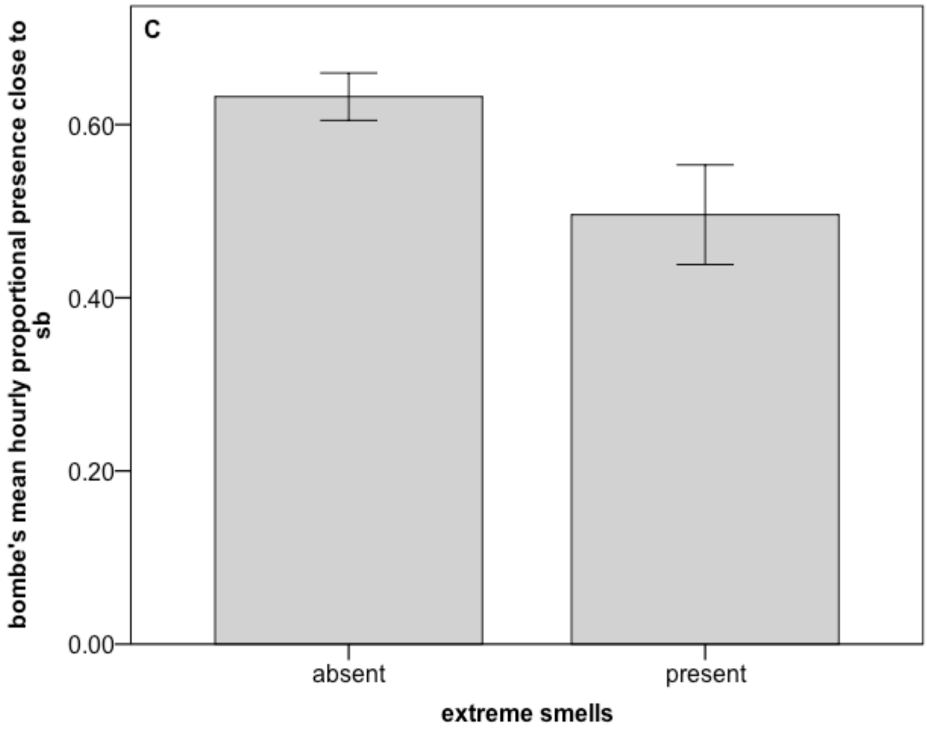

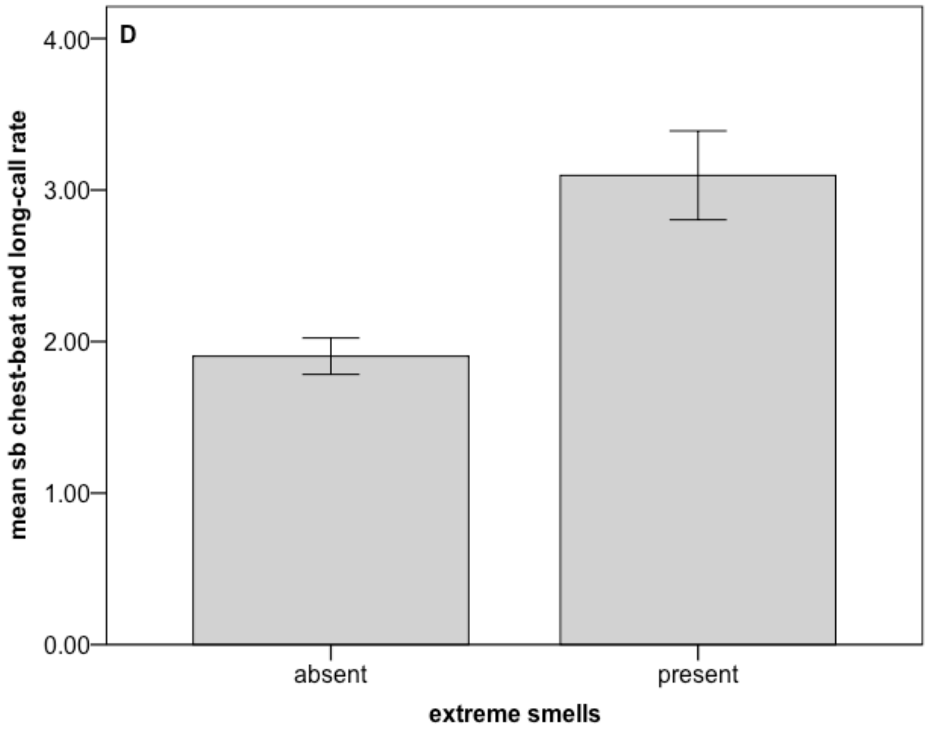

All auditory signals are grouped into hourly rates. For S3A absent *n* = 203, present *n* = 51. For S3B no interaction *n* = 212, interaction *n* = 43. For S3C absent *n* = 145, present *n* = 40. For S3D absent *n* = 203, present *n* = 51. Since interaction presence/absence and extreme smell presence/absence are both categorical variables, Figure S3B axes were reversed for visual purposes so the format of all four graphs could remain similar. Error bars **±** 1s.e.

Figure S4. Additional information for total monthly interactions in relation to silverback extreme smell.

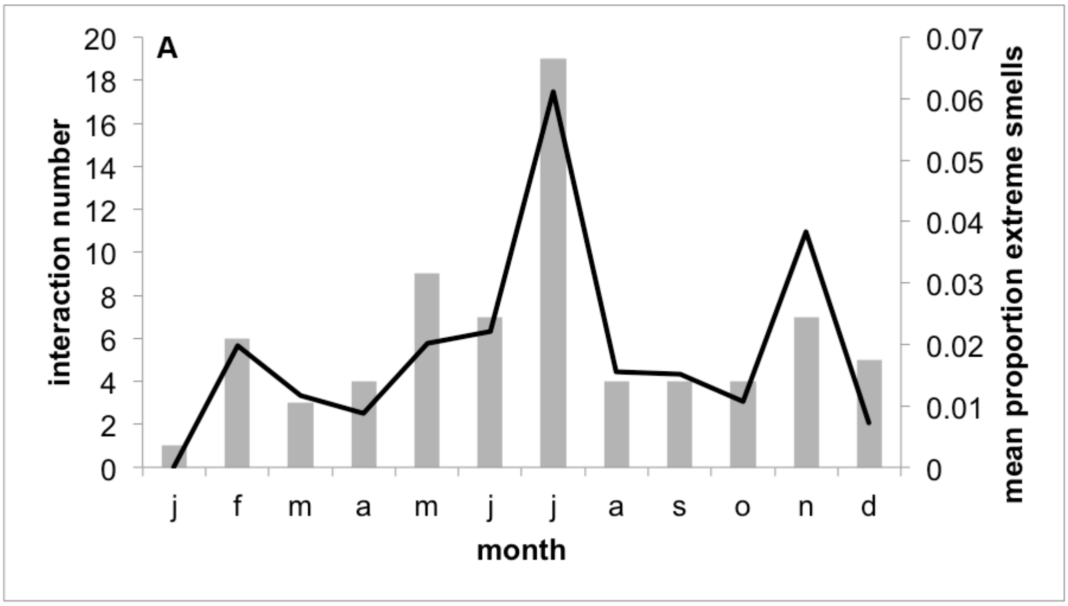

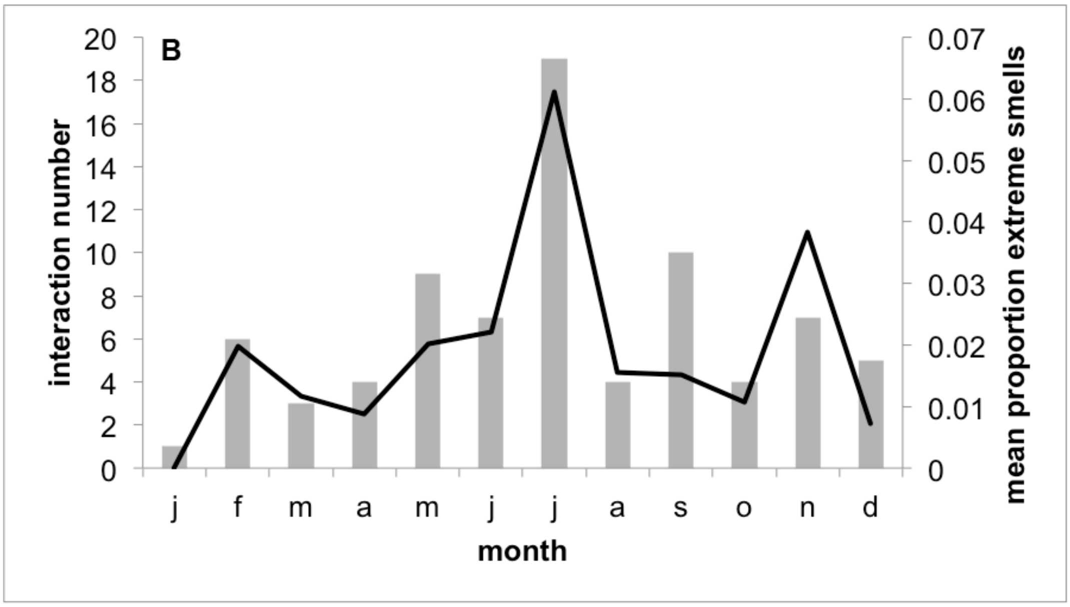

For Figure S4A total number of interactions *n* = 79. For Figure S4B *n* = 6 September interactions that occurred on non-recording days are excluded from the graph as this is the only month where more interactions occurred on non-recording days. If these six data points are not excluded, total interactions per month explain 79% of the montly variance in extreme smells (r = 0.889, p = 0.001, n = 12, controlling for silverback total auditory signals and mean monthly rainfall.)

Supporting Tables

Table S1. The Makumba group birthdates, ages and family trees.

individual	sex	birth & age at start of study period	offspring/mother	age-sex class
makumba	♂	adult silverback	-	mature male
mopambe	♀	adult	bokata, mai, etefi	mature female
malui	♀	adult	tembo, mossoko abuli, mio	mature female
bombe	♀	adult	mobangui, essekerende, silo	mature female
kunga	♂	1998-1999; 8-9 years	mopatapata	blackback
etefi	♀	1999-2000; 7-8 years	mopambe	sub-adult
silo	♂	1999-2000; 7-8 years	bombe	sub-adult
mio	♀	2000-2001; 6-7 years	malui	juvenile
mai	♀	jan 2003; 48 months	mopambe	juvenile
essekerende	♀	mar 2003; 46 months	bombe	juvenile
mossoko abuli	♀	feb 2004; 35 months	malui	juvenile
bokata	♀	jan 10 2006; 12 months	mopambe	infant
mobangui	♂	jul 23-27 2006; 5 months	bombe	infant
tembo	♂	dec 4 2007	malui	infant

Although Etefi was approximately eight years old when data collection commenced, she was not included as an adult female for analysis since her age bordered

between adolescence and adulthood, and unlike the other adult females, she was a presumed natal daughter of the silverback [1, 2, 3].

Table S2. Recorder non-sensitization to silverback smell.

recorder	odor level	β	s.e.	wald	df (predictor and model)	exp(β)	sig
combined recorders	extreme	0.061	0.048	1.597	1,2	1.062	0.206
	high	-0.064	0.041	2.382	1,2	0.938	0.123
	low	-0.075	0.055	1.834	1,2	0.928	0.176
recorder one: sessions recorded *n* = 201	extreme	0.038	0.070	0.294	1,2	1.039	0.888
	high	-0.006	0.062	0.010	1,2	0.994	0.919
	low	-0.064	0.082	0.602	1,2	0.938	0.438
recorder two: sessions recorded *n* = 57	extreme	0.438	0.403	1.180	1,2	1.550	0.277
	high	0.253	0.307	0.708	1,2	1.288	0.400
	low	-0.454	0.450	1.015	1,2	0.635	0.314

Three sessions were omitted from this analysis due to missing information. Odor level was the outcome variable and month (continuous) was the predictor variable, and the monthly number of inter-unit interactions were controlled for in the analysis (n = 255).

Table S3. Data recorded during instantaneous scans and *ad libitum* smell ratings.

**scan data collection categories**	**definition**
silverback position	sit, stand, lying down
silverback behavior	feed, move, rest (including social)
silverback visual monitoring of observers[a]	ignore, low, medium, high
distance of recorder to the silverback	nearest meter
silverback height	tree, ground
number of ‘neighbors within five meters’ of the silverback (hereafter ‘neighbors’)	
identification of neighbors to the silverback	
distance of neighbors to the silverback[b]	nearest meter
neighbor position, behavior and height	same as for silverback above
individuals within 6-10 meters of the silverback	presence, absence
group activity	feed, move, rest (including social), mixed; defined as the majority activity observed where at least two individuals other than the silverback must have been present
forest density[c]	dense, moderate, open
forest zone[d]	*Marantaceae* and *Affromomum* spp., riverine

	(*Raphia hookeri*), clearing, mixed, primary (*Gilbertiodendron dewevrei*), transition
silverback location in his group of neighbors	periphery, middle
silverback location within the entire group	front, middle, back
group spread[e]	close, midrange, dispersed
wind[f]	still, windy
number of tourists	
number of researchers and trackers	
location on map of group's range	location marked within every one 500 meter x 500 meter quadrat; each quadrat was further subdivided into nine 166 meter x 166 meter plots and location was marked within one of these plots
maximum temperature (celsius) and rainfall (millimeters)	recorded at the end of each day for the entire study period
smell	none, low, high. extreme

[a]Defined as (1) ignore, silverback was unaware of human presence or was not paying observers any attention (i.e. feeding with back turned); (2) low, silverback made occasional glances towards observers but continued with his activity (i.e. observer team approached him and he looked our way initially but then continued with the activity he was performing prior to our advance); (3) moderate, silverback still continued with the activity he was performing prior to our advance, whilst keeping a keen eye on the observers (i.e. regular glances and soft barks which represent mild aggression or warning signals); (4) high, silverback was constantly

monitoring observers whilst paying little attention to his former activity (i.e. barked or charged observers and was visibly very uncomfortable with human presence).

[b]Exact distances from the silverback to an individual in a tree were recorded (i.e. base of tree was not used as a distance category from the neighbor).

[c]Defined as (1) dense: a minimum of 75% of the body of a gorilla could only be seen no further than 0-5 meters away, (2) moderate: a minimum of 75% of the body of a gorilla could only be seen no further than 6-10 meters away, (3) open: a minimum of 75% of the body of another gorilla could be seen further than 11 meters away.

[d]Note that open forest density environments include clearings and monodominant forests, such as *Gilbertiodendron dewevrei* forests. Moderate forest density environments include semi-deciduous forests which house a wide diversity of species varying in undergrowth density. Dense forest environments include thick riverine (*Raphia hookeri*) habitats and secondary growth forests dominated by young trees, saplings and herbs such as *Marantaceae* and *Affromomum* spp.

[e]Defining group spread has always been a topic of debate and is not easy to calculate for western lowland gorillas due to their large potential group spread (> 500m), which necessitates several research groups to follow different parts of the group and the possible sub-groups that can occur during feeding. Since most western lowland gorilla groups contain only one adult silverback, it is likely that major changes in group dynamics will be mirrored in the behavior of the adult male since he is the 'protector' of the group. Defining group spread based on the number of individuals within close range of the silverback should accurately reflect changes within group cohesiveness. Therefore, group spread was categorized as (1) close, seven or more individuals within 20 meters of the focal silverback; (2) midrange, 4-6 individuals within 20 meters of the focal silverback on the ground or within 50 meters of the silverback when in a tree; (3) dispersed, less than or equal to three individuals

within 20 meters of the focal silverback. The 50 meters category was only used in the midrange definition because there were times were individuals were high in the canopy but still within hearing and visual presence of both Makumba and the researcher. When the group was dispersed, no more than three other group members were heard or seen.

[f]Defined as the presence of a breeze or a strong wind.

Table S4. Additional information on data groupings used in analysis.

data	grouping justification
observer-silverback distance	median observer-silverback distance scores were calculated to control for the times when the silverback was in a tall tree and thus unlikely to be affected by human presence or direct aggression towards observers
research team, tourist, total observer numbers	mean numbers were calculated and corrected for the number of minutes that different human group sizes were present; means were used due to the absence of outliers
silverback position, behavior and height, group activity, neighbor positions and behavior, forest zones	modal scores were calculated as these data are purely categorical

wind	(a) if wind was recorded in any scan for a session, that session was coded as 'windy' (*n* = 133 sessions) and where no wind was noted, the session was coded as still (*n* = 125); sessions were either quite windy or very still, thus this grouping represented the conditions most accurately
auditory signals<br>.	a mean hourly rate relative to the number of minutes of observation in each session was calculated for all auditory signals; auditory signals were only analyzed by session to minimize the effects of dependence
forest density, silverback visual monitoring of observers, number of neighbors to the silverback (five meters)	mean scores were calculated to (a) correct for varying observation minutes, and (b) allow for a standardized and comparable measure across categories
group spread	proportion of each group spread type (e.g. close, dispersed) was calculated out of total group spread recordings for each session; proportions were used to ensure that certain group spread ratings were not artificially suppressed as may have been the case had rates been calculated

Table S5. Field note descriptions of quiet and loud silverback responses during high intensity interactions.

date	interaction type	description
7/6	loud	The group was sleeping, Makumba walked off and then screaming from the adult females began. Makumba raced back and we ran off after him. Makumba ran towards the solitary male and started chest-beating, tree breaking, and strutting. The group had already moved off at this point, but Makumba kept returning to the solitary male, chest-beating, displaying and then running off, only to return again.
7/7	quiet	We bumped into the group while they were eating. They were dead silent. They all moved very cohesively and quietly. There was no play, no vocalizations, and when Makumba belched they were 'whisper-like' belches.
7/8	quiet	We kept walking in circles trying to find them, knowing that the group was close by but they were not making a sound. We found them when Makumba 'silent' charged us by jumping out of the bushes, staring us down. Until mid-afternoon it was silent apart from several 'whisper-like' belches. At one point we even bumped into Malui (*who charges humans often*) and she just sort of jumped, looked at us and quickly walked off.
7/9	loud	We started hearing chest-beating and screaming and we saw diarrhoea and smelled a strong gorilla odor, although I think it was coming from the solitary male as it was an unfamiliar smell. We heard a lot of screaming. Then Makumba showed

		up, looked at us and then angrily ate herbs (displayed using food) while staring at the solitary silverback who was about 10 meters away. We lost Makumba while trying to catch up to him, but found him after, hooting and chest–beating, and several individuals were in trees (definitely Mopambe, Mai, Malui and Mossoko Abuli). It was very silent again at the end of the afternoon. We kept running into elephants everywhere probably because the group was being so quiet and not alerting the elephants to their presence.
7/10	loud	We ran into the group quite quickly. The group didn't seem more spread out today but yet Makumba was whinnying a lot! Makumba appeared to be returning to the first interaction site area, at which point he started chest-beating and whinnying more, even when his group was close. The juveniles were hooting as well.
7/13	loud	Once the solitary male started chest-beating, Makumba started chest-beating and left the group to patrol. He would return to the group and then leave again, and the group remained very silent throughout. Makumba water displayed by chest-beating in the water.
7/15	quiet	When we heard the solitary male scream very near to us, our group hid in the bushes and went dead silent apart from one 'whisper-like' belch from an unknown individual, and hid in the bushes. Kunga patrolled silently.
8/23	loud	Once the interaction started, Makumba chest-beat, displayed and moved his group out of the area quickly. Once the group

		had moved out of the area, whenever he heard the solitary male, he would display, strut, chest-beat, tree break and violently strip leaves from saplings while the group moved off again.
9/10	loud	When the solitary male started screaming, Makumba ran towards the male and started hooting and chest-beating, while the group hid in a *Marantaceae* patch and remained silent. Then Makumba hooted and chest-beat, and all of the group walked out of the *Marantaceae* patch, passed us by single file and re-joined Makumba.
9/12	loud	Everything was going pretty normally until we heard a scream from a juvenile and Makumba bolted in the direction of the scream. Bombe and another juvenile who were with Makumba then bolted in his direction. The whole group then moved very quickly. We heard and saw a confused group, making panicky bark noises and then the group moved quickly east while Makumba stayed behind, chest-beating, strutting and tree breaking. He then ran off quickly to catch up with the group. When we caught up to Makumba and his group, they were moving single file out of the area.
10/16	quiet	The interaction began when we heard one extra-group male chest-beating in one direction, then another from another direction. Makumba got up and immediately got the group together who remained very quiet and moved off. We continued hearing chest-beats from the extra-unit males but nothing from Makumba, while he moved off with the group and

	guarded any individuals trying to veer in the direction from where the extra-group chest-beats were heard.

Table S6. Predictors included in initial forward stepwise regressions.

**predictors**	
1. daily rainfall	21. adult female human directed aggression
2. daily maximum temperature	22. juvenile human directed aggression
3. silverback-human monitoring	23. grouped observer-silverback distance
4. group spread	24. mopambe roll call
5. corrected forest density	25. bombe roll call
6. corrected number of neighbors (within five m) to silverback	26. malui roll call
7. silverback anger and distress	27. kunga roll call
8. silverback excitement	28. etefi roll call
9. silverback long-call and chest-beat	29. mio roll call
10. silverback soft	30. silo roll call
11. adult female anger and distress	31. mai roll call
12. adult female excitement	32. mosoko abuli roll call
13. adult female long-call and chest-beat	33. essekerende roll call
14. adult female soft	34. interaction presence or absence (category)
15. juvenile anger and distress	35. wind presence or absence (category)
16. juvenile excitement	36. silverback behavior (category)
17. juvenile long-call	37. silverback position (category)
18. juvenile soft	
19. juvenile hand-clap and chest-beat	
20. silverback human directed aggression	

Each category in a categorical variable represents one predictor variable thus a total of 45 predictors could be included in initial Forward Stepwise Logistic Regressions. Due to the high correlation (r > 0.800) between interaction presence/absence and

interaction level, models were run for both predictors separately. If measured conservatively at four data points for every loss in degree of freedom, the model would require a minimum sample size of n = 180, thus sample sizes fall within that necessary for analysis of 45 predictors.

Table S7. Predictors of low silverback (sb) smell.

predictors	β	s.e.	wald	df	model r^2 at each step	exp(β)	sig
constant	4.759	0.794	35.952	1	-	116.634	< 0.001
corrected forest density score	-1.913	0.515	13.803	1	0.068	0.148	<0.001
corrected number of nearest neighbors (5 meters) to sb score	1.939	0.857	5.116	1	0.107	6.951	0.024
wind presence	-1.381	0.554	6.217	1	0.184	0.251	0.013
mio hourly presence	-1.093	0.474	5.307	1	0.247	0.335	0.021

Logistic regression where overall model X^2= 26.964, df = 4, R^2= 0.247, p < 0.001, n = 186; 66 sessions omitted from analysis due to the inclusion of roll call as a predictor in the logistic regression. Unlike extreme odor, which was not influenced by environmental factors, low level smells were most highly influenced by environmental factors. Environmental factors that mask detection of low smells used in close intra-unit communication could be beneficial, making it difficult for extra-unit males to eavesdrop on group social dynamics [4].

Supporting References

1. Klailova M (2011) Interunit, environmental and interspecific influences on silverback-group dynamics in western lowland gorillas *(Gorilla gorilla gorilla)*. PhD Dissertation. Scotland: University of Stirling. 367 p.

2. Breuer T, Hockemba BNM, Olejniczak C (2009) Physical maturation, life-history classes and age estimates of free-ranging western gorillas – insights from Mbeli Bai, Republic of Congo. Am J Primatol 71: 106-119.

3. Parnell RJ (2002) Group size and structure in western lowland gorillas (*Gorilla gorilla gorilla*) at Mbeli Bai, Republic of Congo. Am J Primatol *56*: 193-206.

4. Gosling LM, Roberts SC (2001) Scent marking by male mammals: cheat-proof signals to competitors and mates. Adv Stud Behav 30: 169-217.
